# The Spatiotemporal Dynamics of Phase Synchronization during Epileptogenesis in Amygdala-Kindling Mice

**DOI:** 10.1371/journal.pone.0153897

**Published:** 2016-04-21

**Authors:** Jia-Jia Li, Yong-Hua Li, Hai-Qing Gong, Pei-Ji Liang, Pu-Ming Zhang, Qin-Chi Lu

**Affiliations:** 1 Department of Neurology, Ren Ji Hospital, School of Medicine, Shanghai Jiao Tong University, 160 Pujian Road, Shanghai 200127, China; 2 School of Biomedical Engineering, Shanghai Jiao Tong University, 800 Dongchuan Road, Shanghai 200240, China; University of Modena and Reggio Emilia, ITALY

## Abstract

The synchronization among the activities of neural populations in functional regions is one of the most important electrophysiological phenomena in epileptic brains. The spatiotemporal dynamics of phase synchronization was investigated to reveal the reciprocal interaction between different functional regions during epileptogenesis. Local field potentials (LFPs) were recorded simultaneously from the basolateral amygdala (BLA), the cornu ammonis 1 of hippocampus (CA1) and the mediodorsal nucleus of thalamus (MDT) in the mouse amygdala-kindling models during the development of epileptic seizures. The synchronization of LFPs was quantified between BLA, CA1 and MDT using phase-locking value (PLV). During amygdala kindling, behavioral changes (from stage 0 to stage 5) of mice were accompanied by after-discharges (ADs) of similar waveforms appearing almost simultaneously in CA1, MDT, as well as BLA. AD durations were positively related to the intensity of seizures. During seizures at stages 1~2, PLVs remained relatively low and increased dramatically shortly after the termination of the seizures; by contrast, for stages 3~5, PLVs remained a relatively low level during the initial period but increased dramatically before the seizure termination. And in the theta band, the degree of PLV enhancement was positively associated with seizure intensity. The results suggested that during epileptogenesis, the functional regions were kept desynchronized rather than hyper-synchronized during either the initial or the entire period of the seizures; so different dynamic patterns of phase synchronization may be involved in different periods of the epileptogenesis, and this might also reflect that during seizures at different stages, the mechanisms underlying the dynamics of phase synchronization were different.

## Introduction

Recent structural and metabolic imaging studies, such as functional magnetic resonance imaging, have demonstrated that an epileptic network comprises anatomically and, more importantly, functionally connected cortical and subcortical regions [1, 2]. Epilepsy, including focal epilepsy, requires the transient integration of numerous functional regions distributed over the brain [3]. The idea that the hyper-synchronous activity of pathological assemblies of neurons is the hallmark of epileptiform activity has long been a central notion of the electrophysiology of the epileptic brain [4, 5]. Furthermore, the inhibition of neuronal synchrony by anticonvulsant agents can effectively suppress seizures [6, 7]. It has been observed that synchronization differs across different spatial scales. For example, during ictal activity, the synchronization was increased among local cortical regions but remained unchanged or even decreased among distant cortical regions [8–10]. However, the mechanism by which synchronization evolves during epileptogenesis is still unknown. It is essential to research the spatiotemporal synchronization evolution underlying epileptogenesis, which may help to explore novel treatment targets for retarding the progression of epilepsy.

It has been supposed that phase synchronization is the most plausible element involved in large-scale integration in the brain [11, 12]. Moreover, phase synchronization features high temporal precision of oscillation and conserves temporal codes [13] and has been adopted as a measure to quantify synchronization between different functional regions [9, 10, 14] and in different frequency bands [15]. The temporal dynamics of phase synchronization between the functional regions during epileptogenesis will be investigated in this study.

Mesial temporal lobe epilepsy (mTLE) is a common type of epilepsy that is characterized by seizures arising in the limbic system. The Papez circuit, which involves the amygdala, hippocampus and thalamus, is one of the major pathways in the limbic system [16]. Several tract-tracing studies have revealed many anatomical connections between the limbic sites (such as the amygdala and hippocampus) and the midline thalamic nuclei [17], and a majority of these connections are excitatory, which may promote the genesis of epilepsy [18]. Moreover, recent studies have suggested that the mediodorsal nucleus of the thalamus (MDT) may have a role in seizure initiation and propagation in mTLE [19–21]. Hence, the dynamic synchronization among the neural activities in these regions (the amygdala, hippocampus and MDT) may contribute to epileptogenesis.

The mouse amygdala-kindling model has been a widely studied animal model of mTLE. In kindling, repeated electrical stimulations lead to a progressive increase in epileptic responses [22, 23]. Because there are serial inductions during the kindling procedure, the model allows for the exploration of the dynamics of neural networks during epileptogenesis.

In the present study, the spatiotemporal dynamics of phase synchronization between functional regions was explored during epileptogenesis. Intra-cerebral local field potentials (LFPs) were obtained from the basolateral amygdala (BLA), Cornu ammonis 1 of the hippocampus (CA1) and MDT in the mouse amygdala-kindling models. Phase-locking values (PLVs) between BLA, CA1, and MDT were analyzed during epileptogenesis. The results were such that different dynamic patterns of phase synchronization were detected in different periods of epileptogenesis. The functional regions were maintained in a desynchronized, rather than a hypersynchronized, state during either the initial or entire period of the seizures, and the phase synchronization enhanced dramatically late in seizures or after their termination. Our findings may be helpful for the understanding of the mechanisms underlying epileptogenesis.

## Materials and Methods

### Animals

Experiments were performed on seven male C57BL/6 mice aged three to five months (SLRC Laboratory Animal Company, Shanghai, China). The mice were housed in individual cages with food and water *ad libitum* and were maintained under a 12 h light/dark cycle. All animal experiments were approved by the Ethics Committee, School of Biomedical Engineering, Shanghai Jiao Tong University (China). All efforts were made to minimize animal suffering and reduce the number of animals used in the experiments. 20 mice were included in this experiment. None of them died before kindling and only one mouse died during kindling.

### Stimulating and recording electrodes

**Fig 1:**
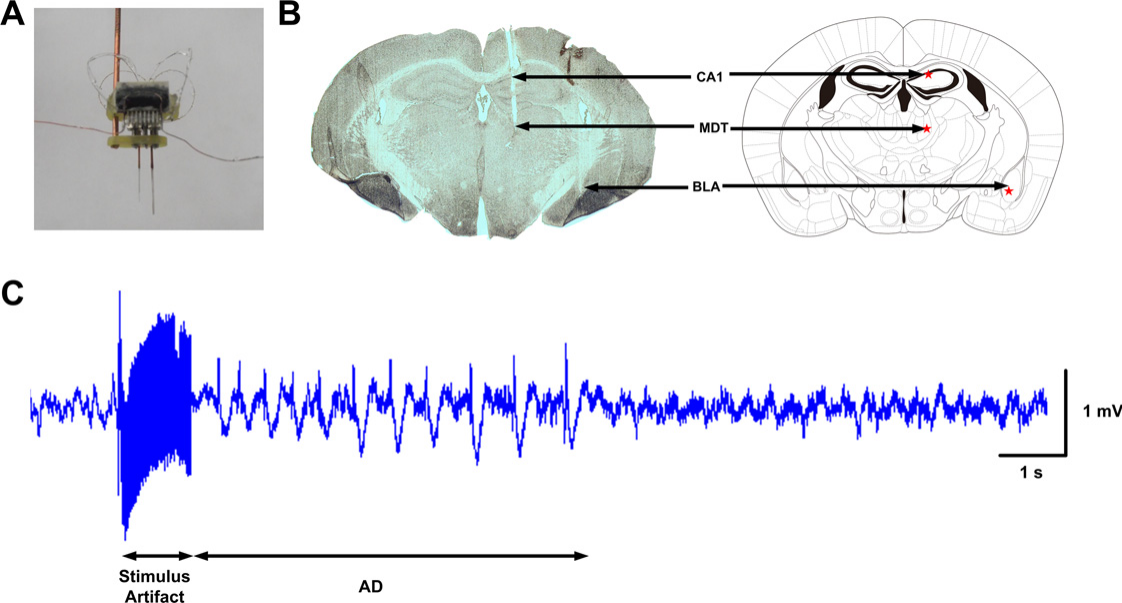
(A) A stimulating and recording microdrive. One 12-pin connector array (for recording electrodes) and one 2-pin header (for stimulating electrode) are glued to the base of a microdrive. Each bundle of two pieces (for one tetrode and one stimulating electrode, or for two tetrodes) of polyimide tubing is glued to an independently movable screw nut on the microdrive base. The free ends of electrode wires are wrapped around to adjacent connection pins. Twelve channels are formatted with three tetrodes. The diameter of the recording electrodes is 13 μm, and the diameter of the stimulating electrode is 50 μm. (B) Histology of one brain slice after kindling and the corresponding atlas (modified from the mouse brain atlas of Franklin and Paxinos [25], Bregma -1.2 mm). The brain slice was stained with Nissl staining (cresyl violet). The stimulating bipolar electrode and one recording tetrode were implanted into the right BLA, and the other two tetrodes were implanted into the right CA1 and the right MDT. Red stars marked the corresponding stimulating and recording sites. (C) A representative recording of the ADs recorded from BLA. The current stimulus train consisted of 1-ms (rectangular square wave) pluses at 60 Hz, and the duration of the stimulus was 1 s. The stimulus artifact resulted from the stimulus train.

The stimulating electrode was a stainless-steel bipolar electrode (50 μm in diameter; A.M. Systems. Inc., Sequim, Washington D.C., USA). The 12-channel recording electrodes consisted of three independent tetrodes. Each tetrode was formed by four twisted polyester-insulated nickel-chrome alloy wires (13 μm in diameter; STABLOHM 675, California Fine Wire Co, USA) with an impedance of 0.5–1 MΩ. All electrodes were secured to a microdrive (Fig 1A), which was constructed as described by Lin et al. [24].

The signals were amplified (×500), filtered (0.5–6,000 Hz), and stored in a computer (16 bits AD converter, 40 kHz sampling rate) using an OmniPlex D Neural Data Acquisition System (Plexon Inc., Dallas, Texas, USA).

### Surgery

The mice were handled for approximately one week prior to surgery to minimize the potential stress of human interaction during the experiments. During surgery, the mice were anesthetized by pentobarbital sodium (100 mg/kg) intra-peritoneally and mounted in a stereotaxic frame (51600, Stoelting Co., Dublin, Ireland, USA). The skin covering the skull was opened, and the skull was exposed and perforated using a high-speed dental drill (K.1070, Foredom Co., USA) with a 1.2 mm diameter drill tip. Seven small holes were drilled, five of which were for the anchor screws, and the other two were for the electrodes. Two screws that were placed in the bilateral frontal bone were used as the ground. The stimulating bipolar electrode and one recording tetrode were implanted into the right BLA (with the bregma as the reference; anteroposterior (AP), −1.2 mm; mediolateral (ML), −2.6 mm; and dorsoventral (DV), −4.9 mm). The other two tetrodes were implanted into the right CA1 (AP, −1.2 mm; ML, −0.6 mm; and DV, −1.7 mm) and the right MDT (AP, −1.2 mm; ML, −0.6 mm; and DV, −3.1 mm) individually according to the mouse brain atlas of Franklin and Paxinos [25].

The microdrive was then fixed to the skull using zinc phosphate cement (Hoffmann Dental Manufaktur GmbH, Berlin, Germany). Throughout the experiments, the body temperature was maintained at 37.5°C using a closed-loop animal blanket system (SS20-2, Huaibei Zhenghua Biologic Apparatus Facilities LTD Co., China). Each animal was allowed at least one week for recovery before the after-discharge (AD) threshold measurement.

### AD threshold measurement and kindling procedure

The electrical stimulation was performed using an isolator (88-302J, Nihon Kohden Co, Tokyo, Japan) and an electronic simulator (SEN-7013, Nihon Kohden Co, Tokyo, Japan) through the stimulating electrode located in BLA. The current stimulus train consisted of 1-ms (square wave) pulses at 60 Hz, and the duration was 1 s. During the kindling procedure, the intensity of current stimuli was firstly set at 60 μA and then increased in steps of 20 μA and delivered at 10-min intervals for the following stimulations to measure the AD threshold. The AD threshold was determined as the current stimulus value by which an AD (as illustrated in Fig 1C), whose duration was no less than 5 s, was detected by the recording electrode in BLA. Subsequently, stimulations at the AD threshold level were administered twice daily between 10 am and 4 pm, with an inter-stimulus interval of no less than 4 h. The behavioral progression of the stimulation-evoked seizures was observed and recorded and was scored according to Racine’s standard classification [26]: stage 0, no behavioral change; stage 1, eye blinking and/or facial clonus; stage 2, head nodding; stage 3, forelimb clonus; stage 4, rearing with forelimb clonus; and stage 5, generalized clonic convulsions with loss of postural control. The fully-kindled state was defined as the occurrence of three consecutive seizures of stages 4~5 [27].

### Histological experiment

At the end of the electrophysiological experiments, the mice were deeply anesthetized with pentobarbital sodium (120 mg/kg), and then perfused transcardially with 20 ml of 0.9% saline solution followed by 50 ml of fixative (4% paraformaldehyde in 0.1 M phosphate-buffered saline (PBS), pH 7.4). The brains were then removed and postfixed in 4% paraformaldehyde in 0.1 M PBS solution at 4°C for 24 h and then equilibrated in 30% sucrose in 0.1 M PBS for at least 12 h. The entire brains were frozen and sectioned coronally on a cryostat microtome set (CM 1950, Leica Co., Wetzlar, Germany) at 50 μm. Signals from the electrodes within the target regions were included in the subsequent data analysis (Fig 1B).

### Data analysis

**Fig 2:**
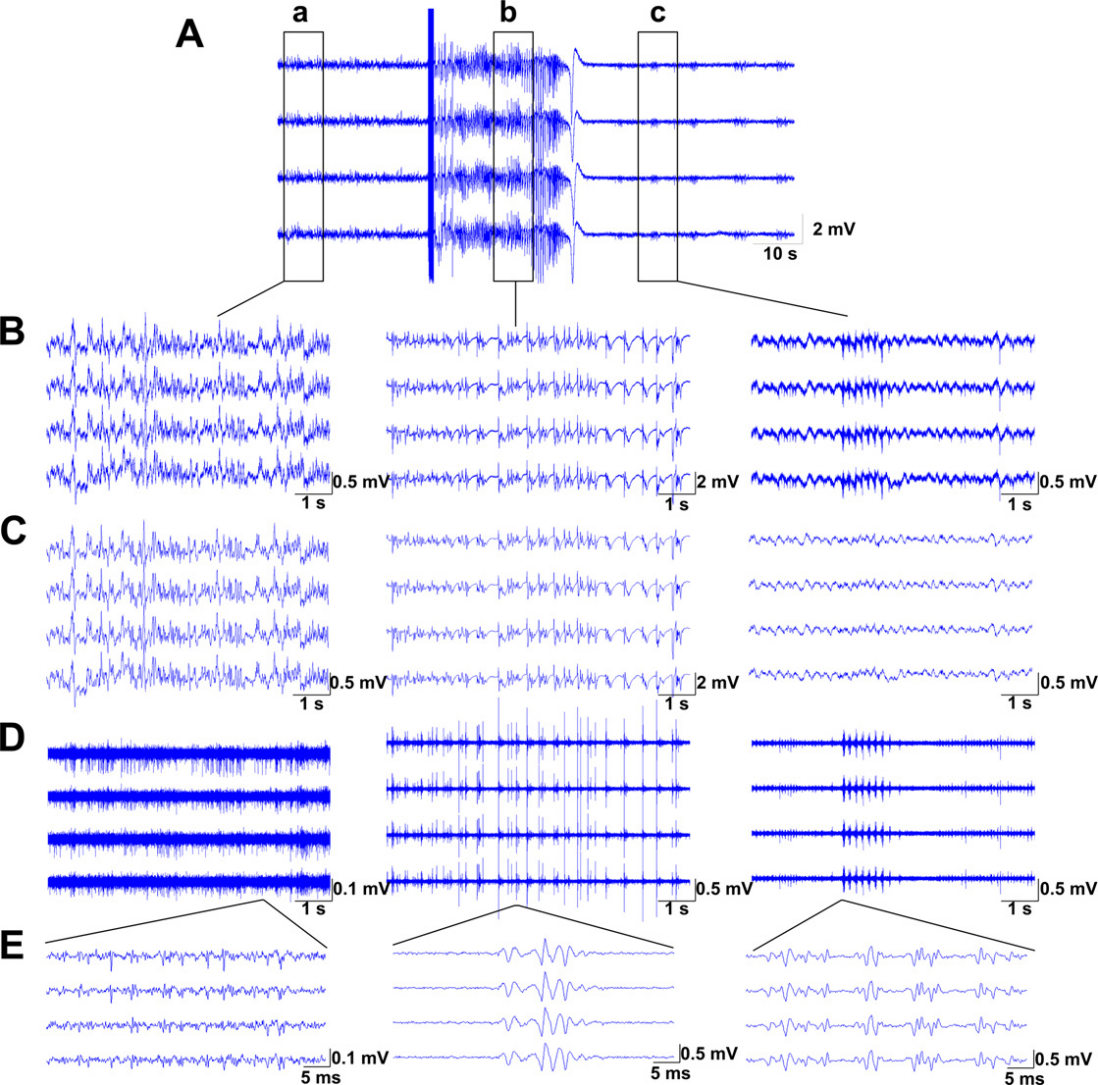
Example of discharges evoked by electrical stimulation. (A) Raw signals recorded in MDT by one tetrode, and the bold blue vertical line indicated the electrical stimulation. (B) The expanded view of signals before stimulation (left column, a), during seizure (middle column, b) and after seizure termination (right column, c). (C) LFPs of corresponding signals by band-pass filtering (0.5–80Hz). (D) MUA of corresponding signals by high-pass filtering (>250Hz). (E) The enlarged view of corresponding MUA in D. It shows that during seizure, the frequency of MUA was higher than that before the stimulation and that after the seizure termination. The variation of the amplitude of MUA showed the same phenomenon.

The raw signals were band-pass filtered (0.5~80 Hz) to obtain LFPs and high-pass filtered (>250 Hz) to obtain multi-unit activities (MUA). An example was illustrated in Fig 2. Because MUA cannot be detected reliably among different regions and different mice in our experiments, only LFPs were investigated in this study. The LFPs were resampled at a frequency of 200 Hz. The LFPs from 4 channels of a tetrode were similar because the distance between the wires in a tetrode was only approximately 15~25 μm. Therefore, we used the signals from one channel to represent the LFPs of the tetrode.

To qualify the phase synchronization between LFPs from BLA, CA1 and MDT, the mean phase coherence (PLV) was calculated [28]. Despite the fact that the definition of phase synchrony can be extended for an arbitrary broad-band signal, a clear physical meaning is available only for narrow-band signals [29]. Thus, the raw signals of each electrode were band-pass filtered to be 4-Hz band signals before calculating the PLV.

The phase value of a signal can be calculated using the Hilbert transform [29], which is defined as

$${\tilde{x}}_{m}(t)=\frac{1}{\pi }\text{P}.\text{V}.\int_{-\infty }^{\infty }\frac{x_{m}(\tau )}{t-\tau }d\tau$$ (1)

where ${\tilde{x}}_{m}(t)$ is the Hilbert transform of time series *x*_*m*_(*t*), and P.V. denotes the Cauchy principal value. The instantaneous phase Φ_*m*_(*t*) of *x*_*m*_(*t*) is calculated as follows,

$$\Phi_{m}(t)=\text{arctan}\frac{{\tilde{x}}_{m}(t)}{x_{m}(t)}$$ (2)

Then, the PLV between two signals *x*_*m*_(*t*) and *x*_*n*_(*t*) is defined as

$$PLV=\frac{1}{N}\left|\sum_{t=1}^{N}\text{exp}\;\left\{j\left[\Phi_{m}(t)-\Phi_{n}(t)\right]\right\}\right|$$ (3)

where Φ_*m*_(*t*) and Φ_*n*_(*t*) are the instantaneous phase of the signals *x*_*m*_(*t*) and *x*_*n*_(*t*), respectively. The PLV ranges from 0 (no phase synchronization at all) to 1 (perfect phase synchronization, *i*.*e*., a constant phase difference within the specified time window).

To investigate the dynamics of the PLVs, a 2-s sliding window with 50% overlap was used, and the PLVs were calculated for pairwise signals and averaged over *N* samples in each window (*N* = 400).

To investigate the phase synchronization in classical EEG frequency sub-bands, the PLVs were averaged over the theta (4~8 Hz), alpha (8~12 Hz), beta (12~32 Hz), and gamma (32~80 Hz) bands in each window.

All data are presented as the mean ± standard deviation. Two-way analysis of variance (ANOVA) was used to analyze the differences among different groups, after which Student-Newman-Keuls (SNK) post-hoc tests were used for comparisons between individual groups. A value of *P* < 0.05 was considered to be statistically significant.

## Results

Neural activities were recorded by using the electrodes shown in Fig 1A from the right BLA, CA1 and MDT of seven mice. Fig 1B shows one example of the histology of brain slices after kindling and the corresponding atlas. Meanwhile, the behavior was also monitored and recorded. An example of the raw signals is shown in Fig 1C. Signals detected from those regions were filtered into LFPs (0.5~80 Hz) and MUA (>250 Hz). A representative example was shown in Fig 2. As illustrated in the figure, during seizures, the frequency of MUA was higher than that before the stimulation and that after the seizure termination. The variation of the amplitude of MUA showed the same phenomenon. Unfortunately, MUA cannot be detected reliably among different regions and different mice in our experiments, so the statistical results were not obtained in this study. In the following study, only LFPs were analyzed.

**Fig 3:**
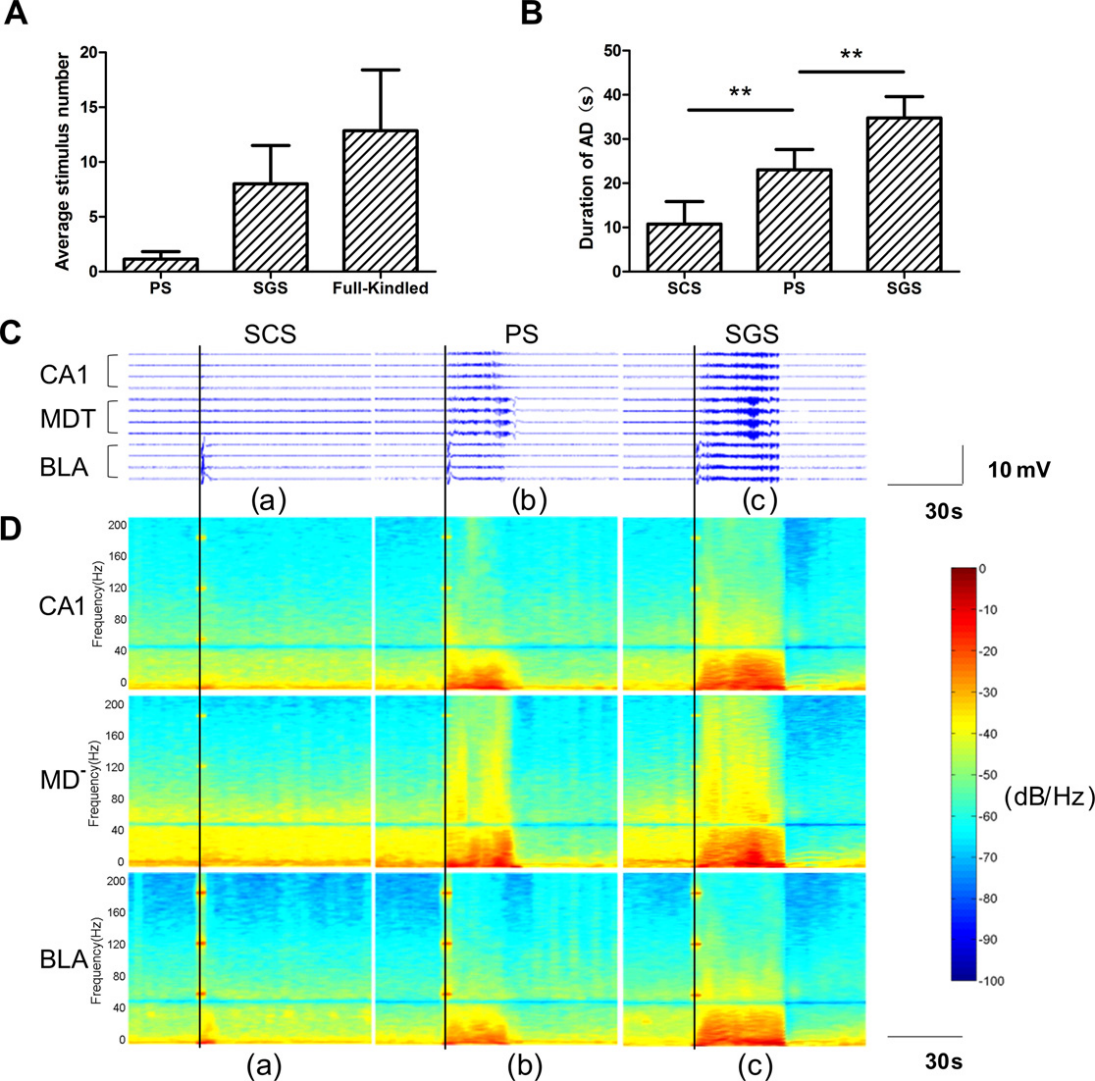
Dynamics of AD during epileptogenesis. (A) The average stimulus number for each stage. (B) Durations of ADs in different stages of epileptogenesis. The durations were positively related to the seizure intensity. Values are expressed as the mean ± SE. (two-way ANOVA, n = 7, ** *P* < 0.01). LFPs and the corresponding power spectrum of an example mouse are shown in C and D. (C) LFPs were recorded from three tetrodes in the CA1, MDT, and BLA during SCS (a), PS (b), and SGS (c). All data were filtered at 0.5~80 Hz. The seizure onset time is marked by the black line, and the seizure termination time is marked by the red line. ADs occurred only in BLA during SCS (a). During PS (b) and SGS (c), ADs in CA1 and MDT, as well as BLA, appeared almost simultaneously, and the waveforms were similar. (D) Power spectrum of LFPs shown in (A). The color bar presented on the right shows the corresponding power per frequency (dB/Hz). There was no significant change in the power spectrum during SCS except for those in BLA (a), where the power mainly increased in the delta and theta bands. During PS (b) and SGS (c), the power increased in the delta, theta, alpha, beta, gamma and even ripple bands, and the power decreased across all frequencies after the seizure termination. The temporal dynamics of power were similar in all three regions. Because of the 60-Hz stimulation, the high power was found at 60 Hz, and its harmonics, at higher frequencies (120 Hz, 180 Hz), during the 1-s stimulation.

The average AD threshold was 160 ± 80 μA (n = 7), and repeated stimulations (Fig 3A, 12.9 ± 5.1, n = 7) were given before the fully-kindled state was reached. There were no behavioral seizures at the beginning of kindling, although ADs were observable, and those seizures at stage 0 [26] were defined as subclinical seizures (SCS) in our present work. After 1~2 stimulations (Fig 3A, 1.1 ± 0.6, n = 7), seizures at stages 1~2 were observed, which were primarily manifested by facial and oral activities, including ipsilateral eye closure, blinking, head nodding and chewing. In our experiments, the seizures at stages 1 and 2 were not necessarily distinguishable and were grouped as partial seizures (PS) [30]. With additional stimulations (Fig 3A, 8.0 ± 3.3, n = 7), seizures at stages 3~5 were observed, which became generalized to bilateral activities and were manifested with bilateral forelimb clonus and rearing that was followed by generalized clonic convulsions with loss of postural control. In the subsequent analysis, those seizures at stages 3~5 were grouped as secondary generalized seizures (SGS) [30]. The durations of ADs were positively related with the seizure intensity (Fig 3B, two-way ANOVA, P < 0.05, n = 7).

### Dynamics of ADs during epileptogenesis

To investigate the spatiotemporal dynamics of network during epileptogenesis, LFP components of the signals, which reflect the summed currents around the synaptic transmission within a local region around the microelectrode tip, were used in the following analysis. Representative LFPs of SCS, PS and SGS from three recording regions in one mouse are shown in Fig 3C. As depicted in the figure, ADs occurred only in BLA during SCS. In the seizures at stages 1~5, ADs were found in all recording regions, and the waveforms were similar. In general, the durations of ADs in all three recording regions were almost equal during the seizures at each stage, although on very rare occasion the AD in MDT persisted for a longer duration than those in the other regions during PS.

For the LFPs shown in Fig 3C, the power spectra were calculated and the results are shown in Fig 3D. Compared with the LFPs before stimulations, there was no significant change in the power spectrum during SCS except for those in BLA, which was the stimulation site, with the power mainly being increased in the delta and theta bands. During PS and SGS, the power was increased in the delta, theta, alpha, beta, gamma and even the ripple bands, and the power was decreased over the wide-band frequency after the seizure termination. The dynamic changes in the power spectrum were similar in all three regions. Because a 50 Hz notch filter was used when collecting the data, it showed a low power at approximately 50 Hz in the power spectrum. These dynamic characteristics of the ADs were also found in other 6 mice.

### Dynamics of PLV during epileptogenesis

Given the above dynamics of ADs, PLV was employed to quantify the dynamics of the phase synchronization between the LFPs recorded from BLA, CA1 and MDT during the kindling procedure.

#### Time of PLV enhancement

**Fig 4:**
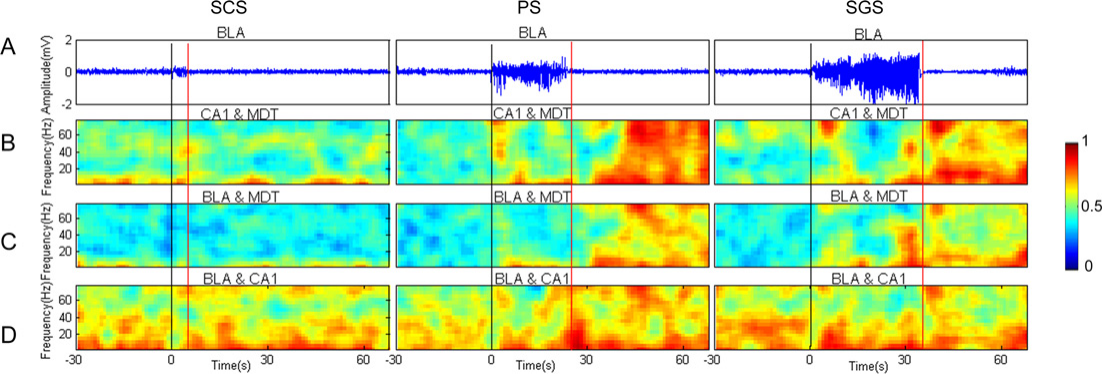
Dynamics of PLV during epileptogenesis in an example mouse. The raw LFPs at different stages of seizures recorded in BLA are shown in (A), whereas (B), (C), and (D) present the corresponding PLVs between CA1 & MDT, BLA & MDT, and BLA & CA1, respectively. The color bar presented on the right shows the corresponding PLVs. The time point 0 represents the seizure onset time and is marked by the black line. The seizure termination time is marked by the red line. During seizures at each stage, the dynamics of the PLVs between LFPs from CA1 & MDT (B) were similar to those from BLA & MDT (C). The PLVs were relatively low and remained approximately unchanged during the entire period of SCS. The PLVs remained at a relatively low level during PS but increased dramatically shortly after the seizure termination across almost all frequency bands and then reached their maximum value. During the initial period of SGS, the PLVs remained at a relatively low level but increased dramatically before the seizure termination across almost all frequencies before reaching their maximum value. The PLVs of the LFPs between BLA & CA1 (D) were maintained at a relatively higher level than those between the other regions during the entire period of the seizure across the three stages.

The temporal dynamic patterns of the phase synchronization during seizures were investigated. The PLVs during each 100-s epoch for SCS, PS and SGS of the example mouse are displayed in Fig 4. Because the LFPs were similar in the three recording regions, the LFPs recorded from BLA are shown in Fig 4A as an example. During the seizures at each stage, the dynamics of PLVs between the LFPs from CA1 and MDT (Fig 4B) were similar to that from BLA and MDT (Fig 4C). The PLVs of the LFPs between BLA and CA1 remained relatively high compared with those between the other regions during the entire period of seizures at the three stages (Fig 4D). Low phase synchronization was observed during the non-seizure condition (before the stimulations). The PLVs were relatively low and remained stable during the entire period of SCS. In PS, the PLVs remained relatively low and dramatically increased shortly after the termination of the seizure. The PLVs reached their maximum value in approximately one second and lasted for approximately 200 seconds before decreasing to the background level. Such PLV dynamics were observed in almost all frequency bands. During SGS, across almost all frequency bands, the PLVs remained at a relatively low level during the initial period but increased dramatically before the seizure ending and reached the maximum value quickly and lasted for about 200 seconds, then decreased to the background level. In the other 6 mice, the observations were similar.

**Fig 5:**
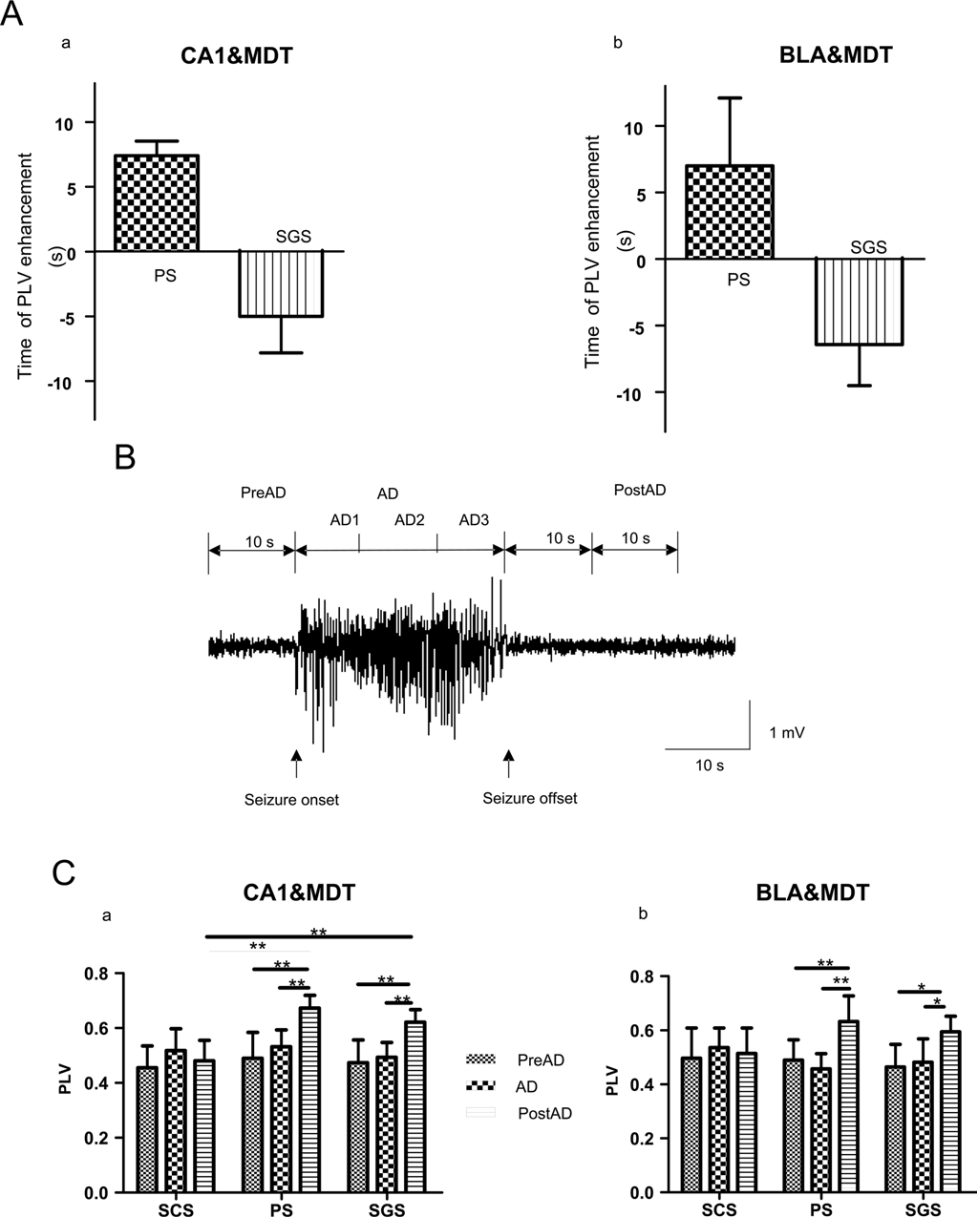
Statistical analysis results of PLVs. (A) Period’s definition. The AD period is the period between seizure onset and termination, and the middle third of the AD period (AD2) was chosen as the representation of AD period. The PreAD period is before the stimulation and was 10-s long. The PostAD period denotes the period after the AD termination and was 10-s long. Statistical analysis results of PLVs between the LFPs from CA1 & MDT are shown in (B), and those from BLA & MDT were shown in (C). During PS, the PLVs of the PostAD were significantly higher than the PLVs of the AD and PreAD (B, C). These results were the same during SGS (B, C). The PLVs of the PostAD between CA1 & MDT during PS and SGS were significant higher than those during SCS (B). Values are expressed as the mean ± SE. (two-way ANOVA, n = 7, * *P*< 0.05, ** *P*< 0.01).

To further investigate the temporal dynamics of the phase synchronization, the time of PLV enhancement was compared between PS and SGS. The time of PLV enhancement was defined as the time point when the PLV exceeded four times the standard deviation from the mean value of a 30-s baseline, which was recorded before the electrical stimulation. The time of seizure termination was set to 0; a negative value of the time point of the PLV enhancement indicated that PLV increased before the seizure termination, whereas a positive value indicated that the PLV increased after the seizure termination. Because the PLVs between BLA and CA1 of different mice did not follow the same dynamics, the results are not included in the statistics. The statistical results are shown in Fig 5A, which confirmed that the phase synchronization between LFPs from BLA and MDT, and those from CA1 and MDT, increased after the seizure termination during PS and increased before the seizure termination during SGS.

#### Dynamics of PLVs

The PLVs between LFPs from different regions were further investigated. Because the seizures initiated in BLA and the durations of the ADs were similar in three regions, the LFPs recorded from BLA were chosen as a reference to define three periods as shown in Fig 5B. The AD period was the time between seizure onset and termination, which was equally divided into three periods, AD1, AD2, and AD3, and the middle section of the AD period (AD2) was chosen as the representation of the AD period. The pre-afterdischarge period (PreAD) denoted a 10-s period before the electrical stimulation. The post-afterdischarge period (PostAD) was chosen as the 10-s period after the seizure termination.

The statistical results of PLVs are shown in Fig 5C. During SCS, the PLVs between each period (PreAD, AD, PostAD) had no significant difference, either between CA1 and MDT, or between BLA and MDT. During PS and SGS, the PLVs of the PostAD were significantly higher than those of the PreAD and AD (two-way ANOVA, *P* < 0.05, n = 7). Furthermore, there was no significant difference between the PLVs of the PreAD and those of the AD. This is also true for the PLVs between CA1 and MDT, and those between BLA and MDT.

Then, the degrees of the phase synchronization in each period (PreAD, AD, PostAD) were compared among the different stages (SCS, PS and SGS). For the signals from CA1 and MDT, the PLVs during the PostAD of PS and SGS were significant higher than those of SCS (Fig 5C, two-way ANOVA, *P* < 0.05, n = 7). No significant difference were found for the other cases.

These statistical results confirmed that the phase synchronization remained unchanged during SCS. During PS and SGS, however, the phase synchronization after the termination of seizure was higher than that before the electrical stimulation. Therefore, the enhanced phase synchronization may be relevant to the seizure termination.

#### Dynamics of PLVs in frequency sub-bands

**Fig 6:**
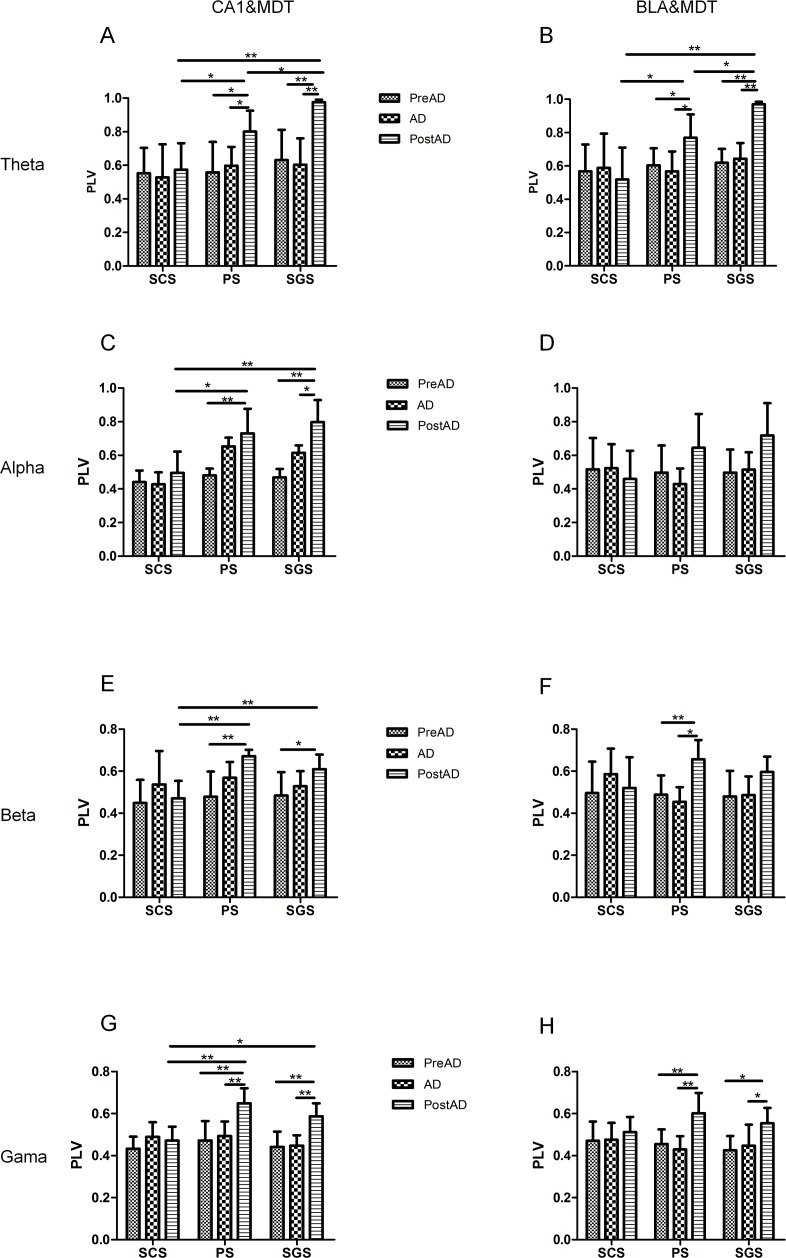
PLVs in different frequency sub-bands between different regions during seizures. Statistical analysis results of the PLVs in the theta (A-B), alpha (C-D), beta (E-F) and gamma (G-H) frequency bands between CA1 & MDT (A, C, E, G), and between BLA & MDT (B, D, F, H). During PS, the PLVs of the PostAD were significantly higher than the PLVs of the PreAD in all sub-bands (A~C, E~H) except in the alpha band between BLA & MDT (D). The PLVs of the PostAD were significantly higher than the PLVs of the AD in all sub-bands (A~B, F~H); except in the alpha and beta bands between CA1 & MDT (C, E), and in the alpha band between BLA & MDT (D). During SGS, the PLVs of the PostAD were significantly higher than the PLVs of the PreAD in all sub-bands (A~C, E, G~H) except in the alpha and beta bands between BLA & MDT (D, F); the PLVs of the PostAD were significantly higher than the PLVs of the AD in theta, alpha and gamma bands between CA1 & MDT (A, C, G), and in the theta and gamma bands between BLA & MDT (B, H).The PLVs of the PostAD during PS and SGS were significantly higher than those during SCS in all sub-bands between CA1 & MDT (A, C, E, G) and in the alpha band between BLA & MDT (B). In the theta band, the PLVs of the PostAD during SGS were significantly higher than those during PS (A~B). The values are expressed as the mean ± SE. (two-way ANOVA, n = 7, * *P* < 0.05, ** *P*< 0.01).

The dynamics of phase synchronization in the theta, alpha, beta, and gamma bands are shown in Fig 6. During SCS, the PLVs between each period (PreAD, AD, PostAD) showed no significant differences in all sub-bands. During PS, there were no significant differences between the PLVs of the PreAD and those of the AD in all sub-bands, and the PLVs of the PostAD were significantly higher than the PLVs of the PreAD in all sub-bands (Fig 6A–6C and 6E–6H), except in the alpha band between BLA and MDT (Fig 6D) (two-way ANOVA, *P* < 0.05, n = 7); the PLVs of PostAD were significantly higher than the PLVs of the AD in all sub-bands (Fig 6A, 6B and 6F–6H) except in the alpha and beta bands between CA1 and MDT (Fig 6C and 6E), and in the alpha band between BLA and MDT (Fig 6D) (two-way ANOVA, *P* < 0.05, n = 7). During SGS, there was no significant difference between the PLVs of PreAD and those of AD in all sub-bands, and the PLVs of the PostAD were significantly higher than those of the PreAD in all sub-bands (Fig 6A–6C, 6E and 6G–6H), except in the alpha and beta bands between BLA and MDT (Fig 6D and 6F) (two-way ANOVA, *P* < 0.05, n = 7).The PLVs of the PostAD were significantly higher than those of AD in the theta, alpha and gamma bands between CA1 and MDT (Fig 6A, 6C and 6G), and in the theta and gamma bands between BLA and MDT (Fig 6B and 6H) (two-way ANOVA, *P* < 0.05, n = 7).

Then, the degrees of phase synchronization in each period (PreAD, AD, PostAD) were compared between each stage (SCS, PS and SGS). The PLVs of PreAD and AD between seizures at different stages in the different sub-bands showed the same results as those shown in Fig 5C. The PLVs of the PostAD during PS and SGS were significant higher than those during SCS in all sub-bands (Fig 6A, 6C, 6E and 6G) between CA1 and MDT, and in the alpha band between BLA and MDT (Fig 6B) (two-way ANOVA, *P* < 0.05, n = 7). There was no significant difference between the PLVs of the PostAD during PS and SGS in the alpha, beta, and gamma bands (Fig 6C–6H); however, in the theta band, the PLVs of the PostAD during SGS were significantly higher than those during PS (Fig 6A and 6B) (two-way ANOVA, *P* < 0.05, n = 7).

These statistical results showed that in all sub-bands, the phase synchronization remained unchanged during SCS. During PS and SGS, in most cases, the phase synchronization after the seizure termination was higher than that before the electrical stimulation. In particular, in the theta band, the PLV magnitude may be a good indicator of the intensity of seizures.

## Discussion

In this study, we investigated the dynamics of the phase synchronization between LFPs from BLA and MDT, and from CA1 and MDT during epileptogenesis using multichannel intracerebral recording in the mouse amygdala-kindling models. We found that the phase synchronization remained approximately unchanged at a relatively low level during the entire period of PS and the initial period of SGS, and that the significant enhancement of the phase synchronization occurred at different time in different stages during the seizures. Meanwhile, the enhancement degree of the phase synchronization during SGS in the theta band was higher than that during PS.

We found that the degree of enhanced phase synchronization in the theta band was positively related to the seizure intensity, but there was no difference between PS and SGS in the other sub-bands (alpha, beta, and gamma). Theta oscillations, which represent the activities of neurons in the hippocampus, can be used to functionally determine the parts of an anatomical macro-system [31]. Therefore, three regions, BLA, CA1 and MDT, all of which belong to the limbic system, were connected more closely, which may be the reason for the phase-locked theta oscillations. Moreover, it was found that increased theta-band functional connectivity could be a hallmark of epilepsy in the mTLE [32]. Theta band oscillations were regulated by GABAergic interneurons, and their discharge frequency could increase during theta oscillations [33]. We speculated that during SGS, the functional areas connected more closely to terminate the seizure, which supports that synchronization may be the cause of seizure termination [34].

An epileptic seizure is traditionally defined as a transient occurrence of signs and/or symptoms due to abnormal excessive or synchronous neuronal activities in the brain. However, the relationship between neural synchronization and seizures is still unclear. In this study, a relatively low level of phase synchronization was found during the entire period of PS and the initial period of SGS. Although it is contrary to the current pervasive presumption that the seizure was a manifestation of excessive abnormal synchronization of neuronal firing in the brain, our result was consistent with those of some recently reported studies. It was reported that the correlation of multichannel EEG either remained unchanged or even decreased during the first half of the seizures in patients with pharmacoresistant focal epilepsy [35]. Another study indicated that LFP synchrony between the seizure focus and the other brain regions was lower in epilepsy patients compared with control patients [10]. The highly heterogeneous neuronal spiking activity during the initiation and spreading of the seizures may contribute to this desynchronization phenomenon, suggesting that there are complex and variable interactions between different neuronal groups during the seizures [36, 37].

A previous study has reported typical, biphasic network dynamics during the preictal state, which is characterized by an early desynchronization phase followed by a late resynchronization phase in animal models induced by pilocarpine, kainate, and picrotoxin [38]. The increased connectivity was observed between the amygdala and the frontal cortex both during the seizures and during the interictal period [39]. It was also found that the synchronization persistently increased before the termination of a focal seizure in patients [40]. In the present study, we also found distinctive network dynamics toward the seizure termination. The phase synchronization remained stable during the preictal and early seizure period but markedly enhanced before the termination of SGS. The increasing synchronization of neural activities before the seizure termination may also be considered as an emergent self-regulatory mechanism for the seizure termination [40]. However, in our experiments, during PS, the synchronization was increased after the seizure termination. Studies have suggested that each stages of epileptogenesis may have its corresponding network composition, which can result in varied brain states [41, 42]. During PS, it seemed that the epileptiform activities propagated from the seizure onset, and reached distant cortical areas with different time delays. Because of the different propagation velocities [35], the synchronization between different regions remained at a low level, not only during the initial period of seizure but also shortly after the seizure termination.

The kindling neuroanatomical studies showed that after developing stage 5 seizures, the destruction of the kindled focus in the amygdala had no impact on the development of the generalized seizures, which were triggered from the sites that were anatomically connected to the amygdala [43]. The study suggested that when the model was kindled to a certain state, the seizures may arise from multiple, distributed cortical micro-domains, and affect cortical regions outside the seizure focus [44]. Consequently, the desynchronization during the initial period of SGS may be ascribed to multiple, distributed cortical seizure onsets.

The time of high phase synchronization in our experiments lasted longer (approximately 200 seconds) than the time of post-ictal depression (approximately 60 seconds), suggesting that the network may continue to integrate even after the seizure termination, which was consistent with our previous study in rat hippocampal slices [45].

Some studies have suggested that MDT may participate in the seizure initiation and propagation in the mTLE [19–21]. One study of patients with focal epilepsy showed that the synchronization between the thalamus and the temporal lobe structures occurred during seizures and that this synchronization tended to be particularly high during the last phase of the seizure [46]. In our study, during SGS, the phase synchronization between the LFPs recorded from BLA and MDT, and those from CA1 and MDT enhanced before the seizure termination. We speculated that MDT may play a role in the seizure termination. However, further pharmacological experiments are warranted to verify this hypothesis.

Amygdala kindling can occasionally lead to spontaneous seizures, but only after long-term kindling, and not in all animals [23]. However, the epileptogenesis not only represents the process of becoming epileptic from a nonepileptic state to the first spontaneous, nonprovoked seizure, but also denotes the process in which seizures slowly increase in duration and recruit additional brain circuits with a subsequent more complex/severe clinical seizure [47]. Therefore, short-term kindling as performed in the present study is a model for epileptogenesis, but not for ictogenesis. It is certainly not justified to equate kindling with complete epileptogenesis in animals that do not have spontaneous seizures. Thus, the results of the current experiment cannot be readily transferred to the condition of human mesial temporal lobe epilepsy.

As shown in Fig 2, during seizures, the frequency and the amplitude of MUA were higher than those before the stimulation and those after the seizure termination. During seizures, more neurons discharged, which might account for this result. Unfortunately, MUA cannot be detected reliably among different regions and different mice in our experiments, and the single-unit activities (SUA) have not been sorted out from those signals by principal component analysis (PCA) method. Several factors may account for this. First of all, each extracellular electrode can record the activities of many adjacent heterogeneous neurons simultaneously with a diameter of 80–100 μm in the brain [48]. Secondly, PCA may be not powerful enough to extract the features of bursting spikes to sort them out [49, 50], which contains a large proportion of overlapping spikes. Thirdly, the relatively low signal-to-noise ratio and high neuronal density of our recording regions, make the separation of distinct units unreliable [51]. So, MUA and SUA had not been analyzed in this study.

Our data showed the dynamics of the phase synchronization during epileptogenesis at the network level and shed light on a deeper understanding of the seizure initiation and termination. From a therapeutic perspective, the role of network synchronization on the seizure termination may have implications for novel antiepileptic or even anti-epileptogenesis treatments.
